# Effects of Cholesterol Supplementation in High Soybean Meal Diet on Growth, Lipid Metabolism, and Intestinal Health of Juvenile Rice Field Eel *Monopterus albus*

**DOI:** 10.1155/anu/2233612

**Published:** 2025-04-04

**Authors:** Kai Xie, Xiang Liu, Yong Shi, Minglang Cai, Jihong Dai, Junzhi Zhang, Yi Hu

**Affiliations:** ^1^Fisheries College, Hunan Agricultural University, Changsha 410128, China; ^2^Hunan Engineering Technology Research Center of Featured Aquatic Resources Utilization, Hunan Agricultural University, Changsha 410128, China

**Keywords:** cholesterol, growth, high soybean meal, intestinal health, lipid metabolism, *Monopterus albus*

## Abstract

The rising cost of fish meal (FM) in aquaculture feed has prompted the search for alternative protein sources like soybean meal (SBM). However, SBM-based diets can negatively affect fish growth, lipid metabolism, and intestinal health. One possible solution is to add cholesterol to SBM-based feeds to mitigate these adverse effects, but the optimal levels and the resulting benefits remain unclear. In this study, the impact of adding cholesterol to low FM and high soybean protein diets on the growth, lipid metabolism, and intestinal health of *Monopterus albus* was evaluated. Juvenile *M. albus* (initial body weight of 20.00 ± 0.02 g) were fed a control diet with 42% FM + 22% SBM diet (FM group), 22% FM + 52% SBM diet (SBM group), and the SBM diet supplemented with 5 g/kg or 10 g/kg cholesterol (SBC5 and SBC10 groups, respectively). The results showed that the weight gain rate (WGR) and hepatosomatic index (HSI) were significantly lower in the SBM group compared to the FM group, but WGR increased with the addition of 10 g/kg cholesterol. Serum alanine aminotransferase (ALT) and aspartate aminotransferase (AST) activities increased significantly in the SBM group, indicating liver stress, but returned to normal levels in the SBC10 group. Cholesterol supplementation also improved serum and liver lipid profiles and significantly increased the contents of total cholesterol (TC) and low-density lipoprotein cholesterol (LDL-C) in serum and high-density lipoprotein cholesterol (HDL-C) in the liver. In addition, cholesterol supplementation increased the activities of intestinal enzymes (e.g., amylase) and restored the structural integrity of the intestinal lining, including villus height and goblet cell count. Additionally, the SBC10 group exhibited a richer and more diverse intestinal microbiota, with increased abundance of Firmicutes and Bacteroidota. These findings demonstrate that supplementing 10 g/kg cholesterol in a high SBM diet improves growth performance, lipid metabolism, intestinal health, and gut microbiota composition in *M. albus*. This study provides a practical food-borne strategy to enhance the use of plant-based proteins in aquaculture while mitigating their negative effects, contributing to the sustainability of fish farming.

## 1. Introduction

Fish meal (FM) stands out as a superior protein source for aquatic animals; however, its scarcity and elevated cost result from the impact of both limited fishery resources and international trade dynamics [1]. Given its noteworthy attributes, including high protein content and favorable palatability, soybean meal (SBM) emerges as the optimal vegetable protein alternative for substituting FM in the diet of fish, particularly carnivorous species [2]. Nevertheless, substituting a high proportion of SBM for FM results in an elevated presence of antinutritional factors such as nonstarch polysaccharides, soybean antigen protein, saponin, and lower levels of cholesterol and certain amino acids in the feed [3, 4]. The research on juvenile redlip mullet (*Liza haematocheila*), seabass (*Lateolabrax maculatus*), and rainbow trout (*Oncorhynchus mykiss*) has indicated that the substitution of FM with SBM leads to a reduction in serum triglyceride (TG) and cholesterol levels, along with decreased lipid deposition in fish [5–7]. Moreover, the high dietary SBM level hinders the digestion and absorption of bait by fish, and some of these factors may induce disorders in intestinal flora, directly impacting the intestinal health of fish [8, 9]. It has been shown that nonstarch polysaccharides in high SBM diets have a high capacity to bind bile salts, lipids, and cholesterol while limiting the physiological role of bile acids and affecting lipid metabolism and cholesterol reabsorption [10, 11]. Therefore, it is of great necessity to solve the decline in growth performance as well as liver and intestinal health problems of aquatic animals caused by high dietary SBM.

Cholesterol, as one of the most important lipids in eukaryotic cells, is essential for the survival of fish [12]. At mechanistic levels, it is responsible for maintaining the strength and integrity of cell membranes. Also, it is involved in the metabolism and transport of animal lipids and acts as a precursor framework for steroid hormones, fat-soluble vitamins, and bile acids [13]. The research findings indicated that supplementing the diet with 10 g/kg cholesterol to diets of Atlantic salmon (*Salmo salar L*.) and hybrid striped bass (*Morone chrysops* × *M. saxatilis*) did not yield a significant impact on growth [14, 15]. Yun et al. [16, 17] found that supplementing a high plant protein diet with 12.5 g/kg cholesterol has been shown to enhance the growth of turbot (*Scophthalmus maximus L*.) and regulate its cholesterol metabolism. Similarly, research conducted on shrimp and crab indicates that supplementing the diet with cholesterol can enhance the growth performance of Pacific white shrimp (*Litopenaeus vannamei*) and Redclaw crayfish (*Cherax quadricarinatus*), boost immune capacity, and positively influence the structure of the flora [18, 19]. The variations in experimental outcomes mentioned above may be associated with differences in species, feed composition, and cholesterol content among aquatic animals.

The rice field eel (*Monopterus albus*) is a freshwater species extensively cultivated in China owing to its high nutritional and economic value, with FM serving as the primary protein source in its compound feed [20]. Currently, given that the protein source constitutes the primary factor in feed cost, there is a growing body of research focused on substituting SBM for FM [21]. Nevertheless, previous studies in our laboratory have revealed that substituting FM with a significant proportion of SBM frequently results in diminished growth performance, disorders in liver fat metabolism, and compromised intestinal health [22, 23]. At present, there are few reports addressing the impact of supplementing exogenous cholesterol to a high SBM diet on the fat metabolism and intestinal health of freshwater carnivorous fish. Hence, the objective of this study was to assess the impact of incorporating two levels of cholesterol (5 and 10 g/kg) into a high SBM diet on the growth performance, fat metabolism, and intestinal health of juvenile *M. albus*.

## 2. Materials and Methods

### 2.1. Ingredients and Experimental Diets

Based on previous research conducted in our laboratory [24], we prepared two diets: a basic diet containing 220 g/kg SBM and a negative control diet containing 520 g/kg SBM. Cholesterol was added to the negative control diet at levels of 5 and 10 g/kg, configured into four experimental groups, FM, SBM, SBC5, and SBC10, all of which were isonitrogenous and isolipidic. The cholesterol was purchased from Shanghai Yuanye Biotechnology Co., Ltd., with a purity of 99%. The protein sources used in the diets included Peruvian steam-dried FM, SBM, brewer's yeast, and corn gluten meal. Fish oil served as the lipid source, while α-starch was used as the carbohydrate source. The FM and fish oil were purchased from Foshan Shunde District Shen Aili Trading Co., Ltd., and the remaining ingredients were obtained from Zhangjiajie Xinrui Biological Feed Co., Ltd. The composition and nutrient levels of the experimental feeds are provided in Table 1. All feeds were air-dried at 25°C to achieve a moisture content of less than 10% and were stored at −20°C. Before feeding, water was added to the feed to form a dough.

### 2.2. Culture Experiment and Management

*M. albus* was obtained from Xihu Farm in Changde, China. A total of 480 *M. albus* individuals, with an initial body weight of (20.00 ± 0.02) g, were randomly assigned to 12 cages (2.0 × 1.5 × 1.5 m) following a 2-week acclimation period and a 24-h fasting phase. Each cage contained 40 fish, with each experimental group having three replicates. In this study, experimental units (such as tanks or animals) were allocated to control and treatment groups using randomization. The randomization sequence was generated using the random number function in Excel. The domestication process involved an initial diet of whole earthworms, followed by a gradual reduction of earthworms and an increase in fish slurry until the diet consisted entirely of fish slurry. Eventually, the fish slurry was replaced by formulated feed. Feed was supplied at an amount equivalent to 3%–5% of the body weight (wet weight) of the fish. The quantity of feed was adjusted on a weekly basis in accordance with the fish's body weight. The feeding was conducted once daily within the time period from 18:00 to 19:00. Hydrological conditions were maintained with a water temperature of 28.5 ± 2.5°C, dissolved O_2_ ≥ 6.5 mg/L, and NH^4+^–N < 0.5 mg/L, respectively. The experiment lasted for 8 weeks. In this study, no unexpected adverse events were observed. This study did not include specific humane endpoints, as no significant distress or adverse health outcomes were anticipated based on prior research and the nature of the procedures.

### 2.3. Sample Collection and Analyses

To minimize potential confounders, the order of measurements for the treatment and control groups was randomized during the measurement process. This was done to avoid any biases or confounding effects that could arise due to the sequence or timing of the measurements. For each experimental group, no animals, experimental units, or data points were excluded from the analysis. All data were included in the final analysis. All experimental protocols were reviewed and approved by the Institutional Animal Care and Use Committee (IACUC) and followed relevant animal welfare guidelines to ensure the humane treatment of animals. After a 24-h fasting period, the number and weight of fish in all cages were measured, and the survival rate (SR), weight gain rate (WGR), and feed conversion rate (FCR) were calculated. Three fish were randomly selected from each cage to determine body length and weight and to dissect the eels, separating the internal organs and liver for weighing. The hepatosomatic index (HSI), viscerosomatic index (VSI), and condition factor (CF) were measured:

SR (%) = 100 × (*N*_t_/*N*_0_),

WGR (%) = 100 × (*W*_t_ − *W*_0_)/*W*_0_,

FCR = *F*/(*W*_t_ − *W*_0_),

HSI (%) = 100 × (*W*_a_/*W*),

VSI (%) = 100 × (*W*_b_/*W*),

CF (g/cm^3^) = 1000 × (*W*/*L*^3^).

where *N*_t_ is the final number of fish; *N*_0_ is the initial number of fish; *W*_t_ is the average final weight (g); *W*_0_ is the average initial weight (g); *F* is the average feed intake (g); *W*_a_ is liver weight (g); *W* is body weight (g); *W*_b_ is visceral weight (g); and *L* is body length (cm).

The intestinal contents from three fish per cage were collected and placed in individually marked 1.5 mL enzyme-free tubes. These samples were promptly frozen with liquid nitrogen and stored at −80°C for future analysis. After a 24-h fasting period, three whole fish from each cage were selected to determine body composition. Additionally, five fish were randomly anesthetized with eugenol (1:12,000, Shanghai, China), and blood was collected via venous puncture using a 2 mL syringe. The blood was then transferred to a 2 mL tube, allowed to stand at 4°C for 6 h, and centrifuged at 3000 rpm for 10 min. The supernatant was carefully collected and aliquoted into 0.5 mL tubes, initially frozen rapidly in liquid nitrogen, and later stored at −80°C for analysis. Concurrently, liver and intestinal tissues were swiftly dissected on an ice tray and placed in 1.5 mL enzyme-free tubes. After freezing, these samples were kept in an ultra-low-temperature freezer for subsequent analysis.

### 2.4. Chemical Analysis

The standard nutrient composition of the feed was evaluated following AOAC (1995) [26] guidelines. The crude moisture content was determined by drying to a constant weight in an oven at 105°C. Crude protein content was assessed using the Kjeldahl nitrogen determination method, while crude fat content was analyzed through the Soxhlet extraction method. Crude ash content was measured using the burning method at 550°C. The method for determining cholesterol in feed is measured by commercial kits (Nanjing Jiancheng Bioengineering Institute). The principle is based on the use of cholesterol oxidase to oxidize cholesterol, generating hydrogen peroxide. Subsequently, under the action of peroxidase, a reaction occurs with the chromogenic agent to produce colored substances. The absorbance of the colored substances at a specific wavelength is measured by colorimetry, thereby quantitatively analyzing the cholesterol content in the samples [27].

### 2.5. Biochemical Indexes of the Serum, Liver, and Intestine

Liver and intestinal samples for the determination of biochemical and fat metabolism indexes were diluted with normal saline at 1:9, centrifuged at 4°C for 3 000 r/min for 10 min, and the supernatant was taken for use. Serum samples were measured directly. Aspartate aminotransferase (AST), alanine transaminase (ALT), blood urea nitrogen (BUN), blood ammonia (BA), acid phosphatase (ACP), serum glucose (GLU), total protein (TP), total cholesterol (TC), TG, nonesterified fatty acid (NEFA), high-density lipoprotein cholesterol (HDL-C), and low-density lipoprotein cholesterol (LDL-C) were quantified using the kit of Nanjing Jiancheng Bioengineering Institute.

### 2.6. Activity of Intestinal Digestive Enzymes

The samples were thawed before determination; the samples for amylase and lipase were diluted with normal saline at 1:9, centrifuged at 4°C for 3000 r/min for 10 min; and the supernatant was extracted for use. The activities of sodium potassium ATPase (NA^+^–K^+^–ATP), amylase, and lipase in proximal intestine and distal intestine digestive were determined by the kit of Nanjing Jiancheng Bioengineering Institute.

### 2.7. Preparation and Observation of Tissue Sections

The distal intestinal tissue was dissected and separated for histological evaluation. The samples were promptly immersed in a paraformaldehyde (4%) solution for fixation. Following a 24 h fixation period, the samples underwent dehydration with conventional alcohol; transparency treatment with xylene, impregnation with wax; and embedding, slicing, and staining with H&E and oil red O staining, culminating in sealing for subsequent observation.

For the analysis of the H&E section of the intestinal tissue, the Scion Image image analysis system was employed. Three nonsequential sections were examined for each sample. In each section, the height of nine villi and muscular thickness were measured, and the number of goblet cells on the villi was counted. Ultimately, the average values from each sample were utilized as the measurement data for statistical analysis.

For the analysis of the oil red O staining section of the intestinal tissue, lipid droplet deposition can be observed using optical microscopy and image analysis. Quantitative results can then be obtained through Image-Pro Plus (version 6.0.0.260).

### 2.8. Intestinal Flora

Bacterial DNA from the posterior intestinal contents of eels was extracted using the QIAamp DNA Stool kit (Qiagen, Hilton, Germany). Following the kit's protocol, impurities such as proteins and RNA were removed to yield purified DNA. The V3–V4 region of the 16S rRNA gene was amplified using the forward primer 338F, 5′-ACTCCTACGGGAGGCAGCA-3′, and the reverse primer 806R, 5′-GGACTACHVGGGTWTCTAAT-3′. The PCR-purified DNA fragments were sequenced on the Illumina platform. Diversity indices—including richness, Shannon, Chao1, Simpson, Bray distance, and Jaccard distance—were calculated using QIIME (v1.9.1) software. Principal coordinate analysis (PCoA) was performed and visualized with weighted gene coexpression network analysis in R software (v2.15.3). Linear discriminant analysis (LDA)-based effect size analysis was conducted using LEfSe software, with a screening value set to ≥2 by default. Taxa abundance at various levels (phylum, class, order, family, genus, and species) among groups was statistically compared using the Metastats Program [28].

### 2.9. Statistical Analysis

Microsoft Office Excel 2019 was employed for the initial sorting and analysis of the experimental data, while statistical software SPSS 25.0 was utilized for comprehensive data analysis. The test statistic in Levene's test adheres to a chi-square distribution. Subsequently, the *F*-test is employed in onne-way analysis of variance to assess whether there exist statistically significant disparities. In case the differences among the groups are significant, Duncan's test is utilized to perform multiple comparisons of the mean values across different treatment groups (*p* < 0.05). All data are presented as means ± standard error of the mean (means ± SEM).

## 3. Result

### 3.1. Growth Performance and Body Composition

Compared with the FM group, the WGR and HSI in the SBM group were significantly decreased, while FCR was significantly increased (*p* < 0.05). Compared with the SBM group, when the cholesterol supplemental level reached 10 g/kg, the WGR was significantly increased, while FCR was significantly decreased (*p* < 0.05). There were no significant differences in SR, VSI, and CF among all groups (*p* > 0.05) (Table 2).

### 3.2. Biochemical Indexes of the Serum, Liver, and Intestine

Compared with the FM group, the activities of serum ALT of *M. albus* in the SBM group were significantly increased (*p* < 0.05), and the contents of serum BA and BUN were significantly decreased (*p* < 0.05). Compared with the SBM group, the activities of serum AST and ALT in the SBC10 group were significantly decreased (*p*  < 0.05), and the contents of BA, ACP, and NEFA in the serum were significantly increased (*p* < 0.05). The activities of AST and ALT in the liver had no significant difference (*p* > 0.05) (Table 3).

Compared with FM group, serum TC content and liver HDL-C content of *M. albus* in SBM group were significantly decreased (*p* < 0.05). Compared with SBM group, supplementation with 5 and 10 g/kg cholesterol significantly increased the contents of TC, LDL-C in serum, and HDL-C in the liver of *M. albus* (*p* < 0.05). In addition, compared with the SBM group, the content of HDL-C in intestine was significantly increased after 5 g/kg cholesterol supplementation (*p* < 0.05) (Table 4).

### 3.3. Intestinal Digestive Enzyme Activity

Compared with FM group, proximal intestinal amylase activity was significantly decreased in the SBM group and SBC5 group (*p* < 0.05) and significantly increased after adding 10 g/kg cholesterol and recovered to the level of the control group. There were no significant differences in proximal intestinal lipase among all groups (*p* > 0.05). Compared with the FM group, there were no significant differences in the activities of sodium potassium ATPase and amylase in the distal intestine of *M. albus* in the SBM group. Compared with the SBM group, the activities of sodium potassium ATPase and amylase in the distal intestine of *M. albus* were significantly increased by adding 5 and 10 g/kg cholesterol (*p* < 0.05) (Table 5).

### 3.4. Intestinal Tissue Structure

**Figure 1:**
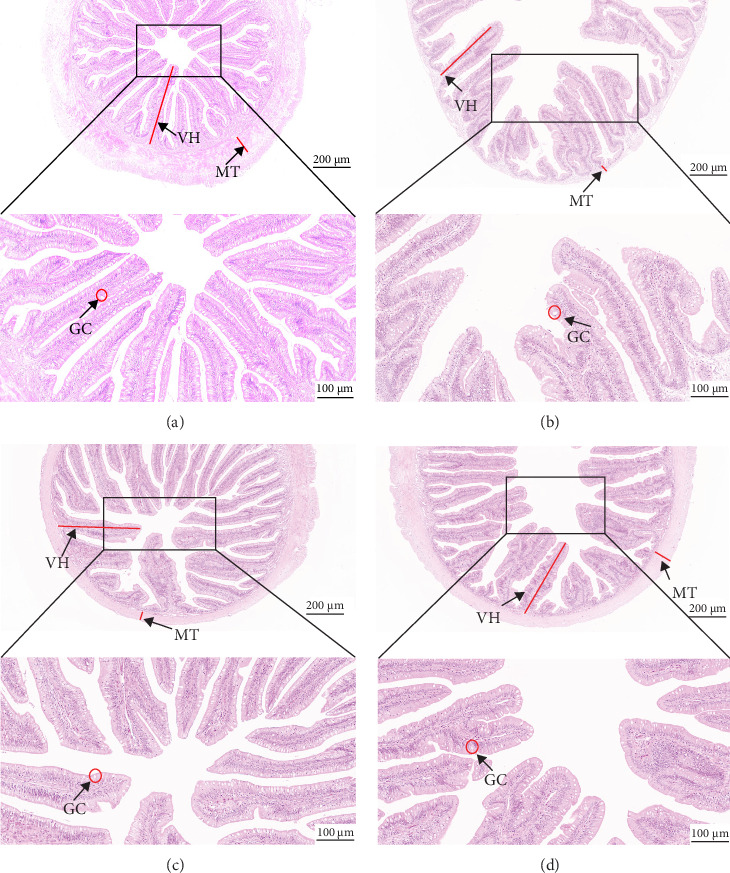
Effects of cholesterol addition in high soybean meal diets on the distal intestinal histology of *M. albus* (H&E staining): (A) FM group (100×, 200×), (B) SBM group (100×, 200×), (C) SBC5 group (100×, 200×), and (D) SBC10 group (100×, 200×). GC, goblet cell; MT, muscle layer thickness; VH, villus height.

**Figure 2:**
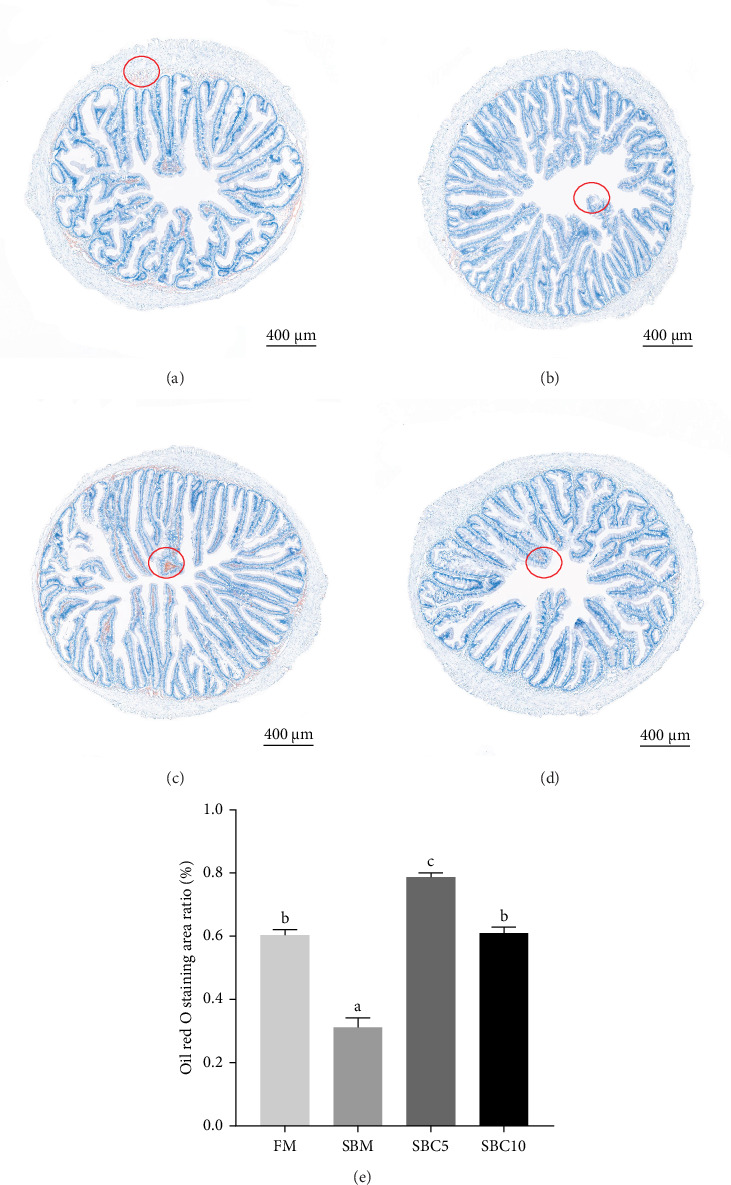
Effects of cholesterol addition in high soybean meal diets on distal intestinal histology of *M. albus* (oil red O staining): (A) FM group (400×), (B) SBM group (400×), (C) SBC5 group (400×), (D) SBC10 group (400×), and (E) oil red O staining area ratio in distal intestinal. In the red circle are fat droplets. Different lowercase superscript letters in the figure indicate significant differences.

As depicted in Figure 1, the replacement of FM with a high proportion of SBM resulted in the crumbling of intestinal villi, detachment of the serous membrane, and thinning of the muscle layer. However, upon the addition of cholesterol to the high SBM diet, intestinal structural integrity was restored, and the villi remained intact.  Table 6 reveals that the intestinal villus height and the quantity of intestinal goblet cells per root in the SBM group were lower than those in the FM group, but there was no significant difference did not exhibit a significant difference (*p* > 0.05). Meanwhile, the muscle thickness was notably lower than that in the FM group (*p* < 0.05). In comparison to the SBM group, supplementation with 5 and 10 g/kg cholesterol significantly increased the villus height, muscle thickness, and goblet cell number in the distal intestine of *M. albus* (*p* < 0.05).  Figure 2 further illustrates that obvious red lipid droplets could be observed in the submucosa of the FM group, and the number of lipid droplets significantly decreased when FM was replaced by a high proportion of SBM. Remarkably, after supplementation with 5 and 10 g/kg cholesterol, intestinal red lipid droplets increased significantly (*p* < 0.05).

### 3.5. Intestinal Flora

#### 3.5.1. Diversity of Intestinal Microflora

**Figure 3:**
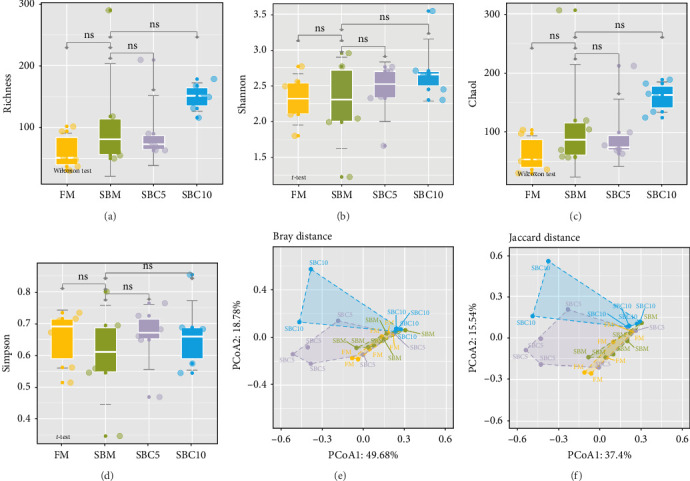
Effects of cholesterol supplementation in high soybean meal diet on the diversity of intestinal microflora: (A) the richness index, (B) the Shannon index, (C) the Chao1 index, (D) the Simpson index, (E) the Bray distance analysis, and (F) the Jaccard distance analysis.

There were no significant differences in richness, Shannon, Chao1, and Simpson among the group, but the richness and Chao1 indexes of SBC10 groups were higher than those of other groups (Figure 3). PCoA revealed variability in intestinal microbiota composition among experimental groups, in which the microbial composition differed in the SBM group from the FM, SBC5, and SBC10 groups (Figure 3E,F).

#### 3.5.2. Dominant Bacteria and Relative Abundance

**Figure 4:**
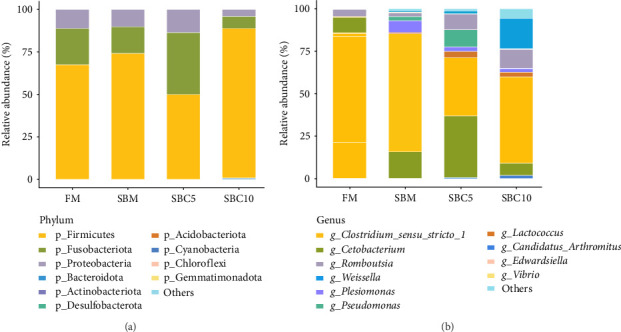
Effects of cholesterol addition in high soybean meal diets in the distal intestinal bacterial indexes of the phylum (A) and genus (B) level.

**Figure 5:**
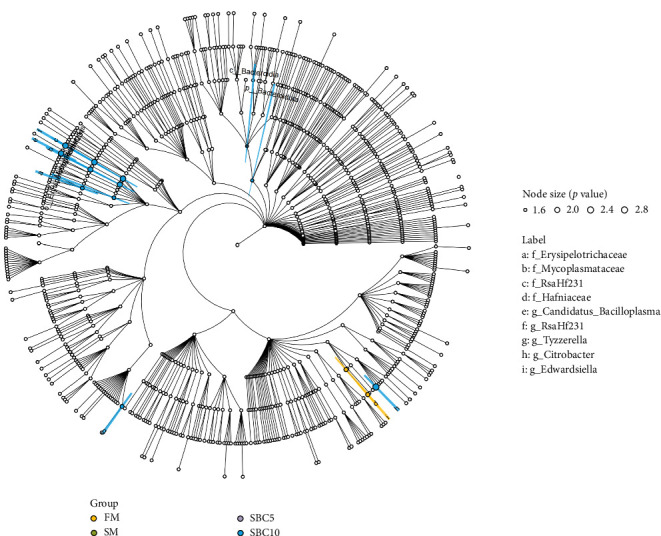
LEfse analysis and phylogenetic cladogram of the gut microbiota of *M. albus*. *Note:* Histogram of LEfSe analysis showing differentially abundant taxa (LDA score ≥2) in the distal intestinal of *M. albus*. The concentric circles represent the taxonomic level from the phylum to species from the inside to the outside. The biomarker of different species is colored according to the group.

As Figure 4A showed, Firmicutes, Fusobacteriota, Proteobacteria, and Bacteroidota were the dominant phyla. Compared with the SBM group, the abundance of Firmicutes and Bacteroidota in the SBC10 group increased. As Figure 4B showed, *Cetobacterium*, *Clostridium*, *Romboutsia* and *Weissella* were the dominant genera. Compared with the SBM group, the abundance of *Weissella* in the SBC10 group increased. From LEfSe analysis, Bacteroidota and *Citrobacter* were the main differentiating bacteria in SBC10 group (Figure 5).

## 4. Discussion

In a previous study employing FM and SBM as the main protein sources, it was found that compared with the low SBM diet (FM: 420 g/kg, SBM: 180 g/kg), the high SBM diet (FM: 320 g/kg, SBM: 330 g/kg) significantly reduced the digestive enzyme activity, impaired the intestinal tissue structure, and consequently hindered the growth performance of *M. albus* [23]. Hu et al. [29] also obtained similar results in the experiment of replacing FM with high SBM of *M. albus*. In this experiment, further increasing the proportion of SBM instead of FM (FM: 220 g/kg, SBM: 520 g/kg) significantly reduced the growth of *M. albus* and decreased the proximal intestine amylase and the distal intestine amylase, lipase, and NA^+^–K^+^–ATP. The distal intestine of the rice field eel is the main site for the absorption and digestion of nutrients. It has more microvilli which are suitable for the colonization of intestinal microorganisms and the fixation of enzymes [30, 31]. Therefore, the distal intestine of the rice field eel was selected for histological observation in this study. The results of distal intestinal section showed that the intestinal villi were broken, the serous membrane was detached, and the muscle layer was thin. However, 5 or 10 g/kg cholesterol supplementation in the high SBM diet can improve growth, enzyme activity, and intestinal red lipid droplets and restore intestinal tissue structure. The results of this experiment are similar to those of Twibell and Wilson [32] in channel catfish (*Ictalurus punctatus*), Yun et al. [16, 17] in turbot, and Deng in rainbow trout; cholesterol supplementation in high SBM diet may improve growth responses in juvenile aquatic animals. Research indicates that antinutrient factors in SBM can interact directly with digestive enzymes in the digestive tract, thereby reducing their activity and affecting the absorption of nutrients [33]. Meanwhile, certain nonstarch polysaccharides exhibit a robust affinity for binding bile salts, lipids, and cholesterol [11]. This binding capacity affects bile acid, leading to a decreased absorption of cholesterol and other lipids, thereby influencing the chylification of fat in the intestine and diminishing lipid digestion and absorption in animals [10]. Therefore, in this experiment, additional cholesterol supplementation can reduce the negative effects of high SBM diet on the growth of *M. albus* to a certain extent.

Fish blood serum indices are closely associated with fish metabolism, nutrient absorption, and disease susceptibility, with its physiological alterations serving as indicators of fish health [34]. Serum ALT and AST levels directly indicate liver injury, while serum BA, BUN levels, and liver ALT and AST levels are closely associated with protein metabolism [35–37]. In this experiment, we found that when FM was replaced with a high proportion of SBM in the diet, the activities of serum ALT and AST of *M. albus* in the SBM group were significantly increased, and the contents of serum BA and BUN were significantly decreased. Compared with the SBM group, the activities of serum AST and ALT in the SBC10 group were significantly decreased, and the contents of BA and NEFA in the serum were significantly increased. It is speculated that replacing FM with high SBM may damage the liver cells of *M. albus*, a hypothesis consistent with previous findings in the laboratory [23, 29]. Liver aminotransferases catalyze the breakdown of amino acids into ammonia, which is subsequently converted into urea. Upon liver damage, a substantial amount of aminotransferase leaks into the bloodstream, disrupting the metabolism of BA and urea nitrogen [38]. The inclusion of 10 g/kg cholesterol in the high SBM diet might enhance the liver's secretion of bile acids and nonesterified free fatty acids, thereby promoting lipid metabolism. This process can also provide intermediary products for the metabolism of other substances such as sugars and amino acids [39].

The content of TG, TC, HDL-C, and LDL-C in serum, the liver, and the intestine was an important index to measure the fish's fat metabolism, especially cholesterol metabolism [40]. In this study, the serum TC content of *M. albus* fed a high proportion of SBM diet decreased significantly, consistent with findings of studies juvenile redlip mullet, seabass, gibel carp (*Carassius auratus* gibelio), and turbot (*S. maximus*) [5, 6, 8, 41, 42]. While vertebrates possess the ability to synthesize cholesterol from sterol precursors, fish fed with high SBM face challenges in the supply of exogenous cholesterol and synthesis of endogenous cholesterol, necessitating the supplementation of exogenous cholesterol [4]. When the high SBM diet supplemented with cholesterol, the serum TC and LDL-C content significantly increased, while the HDL-C contents in liver and intestine significantly increased of *M. albus*. The possible reason is that the supplementation of cholesterol can promote cholesterol metabolism, so that more cholesterol is transported to the liver and further synthesis of HDL-C [43, 44]. In addition, the increase of dietary cholesterol stimulates the liver to produce more HDL by affecting the activity of related enzymes such as cholesterol esterase, thereby alleviating the cholesterol metabolism disorder caused by high SBM and restoring the normal levels of TC in serum and intestine [45, 46]. Interestingly, the results of intestinal oil red also showed that adding cholesterol to the diet helped restore cholesterol homeostasis. This is consistent with the study in turbot that cholesterol supplementation with high SBM diet can restore normal cholesterol metabolism in fish, which is associated with the upregulation of cholesterol synthesis gene cholesterol 7α-hydroxylase [16].

The biological barrier of fish is composed of complex and interrelated microbial communities, and the main microbial composition groups include aerobic bacteria, facultative anaerobic bacteria, and strict anaerobic bacteria [47]. The main composition of the intestinal flora of *M. albus* in this study is consistent with some previous results based on 16SrDNA high-throughput sequencing and metagenomic sequencing [48]. In this experiment, the Chao1 and richness indexes of the intestinal flora of eels in SBC10 group were higher than those in other groups. In addition, the abundance of Firmicutes increased after 10 g/kg cholesterol supplementation. Some studies have shown that Firmicutes are related to fat absorption in the body, and they are the main microbial categories producing short-chain fatty acids, which may be the reason for the improvement of growth performance and the restoration of lipid metabolism of *M. albus* after 10 g/kg cholesterol supplementation [49, 50]. *Weissella* is a member of lactic acid bacteria in probiotics, and it shows certain advantages in *β*-glucosidase activity, antifungal activity, and promoting *Lactobacillus* growth [51]. Some strains of Bacteroidota may produce beneficial bioactive substances such as short-chain fatty acids and antimicrobial peptides that promote the health of fish [52]. *Tyzzerella* has potential probiotic effects in the fish gut microecology, which can improve immune function and enhance the host's absorption of nutrients by regulating the host's immune system [53]. This indicates that 10 g/kg cholesterol supplementation can increase intestinal microbial diversity and improve the relative abundance of beneficial bacteria in the intestine of *M. albus*. Wang et al. [54] found that supplementation with an appropriate amount of cholesterol in grass carp (*Ctenopharyngodon idella*) feed during the middle growth period can reduce the abundance of harmful bacteria in the intestinal and thus resist enteritis after *Aeromonas hydrophila* infection. Li et al. [55] showed that dietary supplementation of cholesterol can regulate hamsters gut microbiota and thus regulate cholesterol metabolism. These results suggest that dietary cholesterol may regulate lipid metabolism of eel by increasing the relative abundance of intestinal beneficial bacteria, enhancing digestive enzyme activity and repairing mucosal epithelial integrity.

## 5. Conclusion

In conclusion, *M. albus* exhibits poor adaptation to a high SBM diet. However, the addition of an appropriate amount of cholesterol to the high SBM diet can counteract the decline in growth performance, mitigate the reduction in digestion and absorption capabilities, alleviate the disorder of lipid metabolism, and ameliorate the intestinal flora induced by a high SBM diet. Under the conditions of this experiment, the suitable cholesterol supplementation level was determined to be 10 g/kg. With the growing interest in plant-based diets for human consumption, understanding how cholesterol supplementation (or dietary cholesterol intake) affects lipid metabolism and gut health could have broader implications.

## Figures and Tables

**Table 1:** Composition of the diets and level of nutrition.

Ingredients (g/kg)	FM	SBM	SBC5	SBC10
Fish meal	420.00	220.00	220.00	220.00
Soy bean meal	220.00	520.00	520.00	520.00
α-Starch	148.00	148.00	148.00	148.00
Microcrystalline cellulose	114.60	0.00	0.00	0.00
Soy oil	15.00	29.60	24.60	19.60
Ca(H_2_PO_4_)_2_	17.00	17.00	17.00	17.00
Choline chloride	5.00	5.00	5.00	5.00
Cholesterol	0.00	0.00	5.00	10.00
Brewer yeast	50.00	50.00	50.00	50.00
^a^Vitamin and mineral premix	10.00	10.00	10.00	10.00
^b^Mold inhibitor	0.30	0.30	0.30	0.30
^c^Antioxidants	0.10	0.10	0.10	0.10
Total	1000.00	1000.00	1000.00	1000.00
^d^Nutrition levels (dry matter [%])				
Crude protein	408.68	404.32	407.44	401.65
Crude lipid	60.66	60.90	61.13	61.45
Ash	102.27	93.67	94.44	94.41
Cholesterol	5.17	3.21	7.99	13.10

^a^Vitamin and mineral premix was provided by MGOTer Bio-Tech Co. Ltd. (Qingdao, Shandong, China), and the specific ingredients can be referred to the previous studies of Xie et al. [25].

^b^The main component of the antioxidant is ethoxyquin.

^c^The main component of the mold inhibitor is calcium propionate.

^d^Crude protein, crude lipid, and ash levels were measured values (dry matter).

**Table 2:** Effects of cholesterol addition in high soybean meal diets on the growth of *M. albus*.

Indexes	Treatments			
	FM	SBM	SBC5	SBC10
IBM (g)	20.00 ± 0.02	20.00 ± 0.03	19.97 ± 0.02	20.00 ± 0.01
FBM (g)	44.34 ± 0.10^c^	36.95 ± 0.38^a^	35.89 ± 0.52^a^	38.45 ± 0.03^b^
WGR (%)	121.70 ± 0.54^c^	84.75 ± 1.74^a^	79.77 ± 2.71^a^	92.25 ± 0.19^b^
SR (%)	85.33 ± 3.53	82.67 ± 1.76	78.67 ± 1.76	82.00 ± 3.06
FCR	1.82 ± 0.03^a^	2.65 ± 0.03^c^	2.89 ± 0.11^d^	2.45 ± 0.04^b^
HSI (%)	4.67 ± 0.30^b^	3.75 ± 0.36^a^	3.17 ± 0.20^a^	3.51 ± 0.28^a^
VSI (%)	21.50 ± 2.03	20.60 ± 1.66	24.50 ± 1.24	23.60 ± 2.08
CF (×10^3^ g/cm^3^)	1.15 ± 0.03	1.15 ± 0.03	1.12 ± 0.04	1.14 ± 0.05

*Note:* Values are presented as means ± SEM. Letters in the same row with different superscripts are significantly different (*p* < 0.05).

**Table 3:** Effects of cholesterol addition in high soybean meal diets on the serum and liver biochemical indices of *M. albus*.

Indexes	Treatments			
	FM	SBM	SBC5	SBC10
Serum				
AST (U/L)	1.22 ± 0.36^ab^	1.79 ± 0.46^b^	1.05 ± 0.3^ab^	0.71 ± 0.15^a^
ALT (U/L)	1.03 ± 0.42^a^	2.61 ± 0.74^b^	0.73 ± 0.22^a^	0.47 ± 0.19^a^
BUN (mmol/L)	10.94 ± 0.21^b^	10 ± 0.25^a^	9.93 ± 0.21^a^	10.36 ± 0.17^ab^
BA (umol/L)	259.09 ± 4.46^b^	234.28 ± 2.33^a^	234.87 ± 8.17^a^	273.01 ± 5.22^b^
ACP (g/L)	4.32 ± 0.2^ab^	4.02 ± 0. 12^a^	4.58 ± 0.15^ab^	4.78 ± 0.29^b^
GLU (mmol/L)	1.97 ± 0.07	1.85 ± 0.17	2.08 ± 0.10	1.84 ± 0.10
NEFA (umol/L)	7.45 ± 0.36^a^	7.45 ± 0.23^a^	19.41 ± 2.4^b^	17.51 ± 0.82^b^
Liver (×10)				
AST (U/gprot)	171.95 ± 6.14	165.18 ± 3.37	165.46 ± 10.59	166.99 ± 13.19
ALT (U/gprot)	293.4 ± 35.89	286.31 ± 18.46	356.39 ± 25.82	367.63 ± 31.82

*Note:* “×10” is the statistical result after multiplying the data by 10 times to better observe the trend change when the value is too small. Values are presented as means ± SEM. Letters in the same row with different superscripts are significantly different (*p* < 0.05).

Abbreviations: ACP, acid phosphatase; ALT, alanine aminotransferase; ALT, alanine aminotransferase; AST, aspartate aminotransferase; AST, aspartate aminotransferase; BA, blood ammonia; BUN, blood urea nitrogen; GLU, serum glucose; NEFA, nonesterified free fatty acids.

**Table 4:** Effects of cholesterol addition in high soybean meal diets on lipid metabolism in the serum, intestine, and liver.

Indexes	Treatments			
	FM	SBM	SBC5	SBC10
Serum				
TG (mmol/L)	0.83 ± 0.08^b^	0.68 ± 0.21^a^	0.63 ± 0.08^a^	0.68 ± 0.06^a^
TC (mmol/L)	1.63 ± 0.13^bc^	1.05 ± 0.18^a^	1.50 ± 0.05^b^	1.94 ± 0.19^c^
HDL-C (mmol/L)	3.02 ± 0.51	2.74 ± 0.60	3.02 ± 0.43	2.71 ± 0.41
LDL-C (mmol/L)	1.33 ± 0.16^ab^	1.26 ± 0.23^a^	1.93 ± 0.20^b^	1.80 ± 0.10^b^
Liver (×10)				
TG (mmol/g)	0.96 ± 0.09	1.05 ± 0.19	1.01 ± 0.13	0.74 ± 0.08
TC (mmol/g)	0.93 ± 0.04	0.59 ± 0.15	0.80 ± 0.09	0.69 ± 0.16
HDL-C (mmol/g)	1.39 ± 0.08^b^	1.15 ± 0.05^a^	1.40 ± 0.07^b^	1.24 ± 0.04^b^
LDL-C (mmol/g)	0.45 ± 0.05	0.43 ± 0.10	0.67 ± 0.07	0.53 ± 0.07
Intestine (×10)				
TG (mmol/g)	0.47 ± 0.05	0.52 ± 0.07	0.38 ± 0.02	0.45 ± 0.06
TC (mmol/g)	0.58 ± 0.06	0.55 ± 0.02	0.62 ± 0.11	0.66 ± 0.19
HDL-C (mmol/g)	1.09 ± 0.05^ab^	1.05 ± 0.06^a^	1.21 ± 0.07^b^	1.08 ± 0.09^a^
LDL-C (mmol/g)	1.34 ± 0.16	0.97 ± 0.05	1.12 ± 0.07	1.28 ± 0.09

*Note:* Values are presented as means ± SEM. Letters in the same row with different superscripts are significantly different (*p* < 0.05).

Abbreviations: HDL-C, high-density lipoprotein cholesterol; LDL-C, low-density lipoprotein cholesterol; TC, total cholesterol; TG, triglyceride.

**Table 5:** Effects of cholesterol addition in high soybean meal diets in the proximal intestine and distal intestine digestive enzyme of *M. albus* (U/gprot).

Indexes	Treatments			
	FM	SBM	SBC5	SBC10
Proximal intestine				
Amylase	269.24 ± 32.52^b^	117.06 ± 5.62^a^	142.54 ± 10.33^a^	250.65 ± 14.68^b^
Lipase	159.91 ± 49.65	165.17 ± 47.51	189.24 ± 68.85	170.9 ± 78.79
Distal intestine				
Amylase	321.62 ± 10.38^ab^	299.03 ± 17.49^a^	354.71 ± 13.14^b^	352.58 ± 20.1^b^
Lipase	171.5 ± 51.99	88.35 ± 24.6	149.33 ± 36.27	209.85 ± 72.4
NA^+^–K^+^–ATP	18.17 ± 2.36^ab^	15.04 ± 2.02^a^	22.87 ± 1.57^b^	30.6 ± 1.24^c^

*Note:* Values are presented as means ± SEM. Letters in the same row with different superscripts are significantly different (*p* < 0.05).

**Table 6:** Effects of cholesterol addition in high soybean meal diets on the intestinal histology of *M. albus*.

Indexes	Treatments			
	FM	SBM	SBC5	SBC10
VH (μm)	571.45 ± 25.29^ab^	549.05 ± 24.86^a^	681.48 ± 26.52^c^	632.9 ± 24.43^bc^
MT (μm)	93.95 ± 2.03^c^	18.78 ± 2.98^a^	66.33 ± 5.42^b^	84.20 ± 5.2^c^
GC (N)	47.5 ± 5.38^a^	38.75 ± 2.56^a^	78.99 ± 3.72^b^	67.75 ± 2.78^b^

*Note:* Values are presented as means ± SEM. Letters in the same row with different superscripts are significantly different (*p* < 0.05).

Abbreviations: GC, amounts of intestinal goblet cells per root; MT, intestinal muscular thickness; VH, intestinal villus height.

## Data Availability

The data that support the findings of this study are available from the first author upon reasonable request.
